# Criterion Validity and Inter-Method Reliability of a Smartphone Sensor-Based Application for Lower-Limb Range of Motion: In-Person vs. Tele-Assessment

**DOI:** 10.3390/s26051661

**Published:** 2026-03-06

**Authors:** Rehab Aljuhni, Zainab Aldarwish, Shroug Almutairi

**Affiliations:** Department of Physical Therapy and Health Rehabilitation, College of Applied Medical Sciences, Majmaah University, Al Majmaah 15341, Saudi Arabia

**Keywords:** tele-rehabilitation, smartphone sensors, range of motion, remote assessment, mobile health applications, musculoskeletal monitoring

## Abstract

**Highlights:**

- The PhysioMaster application showed strong validity and inter-method reliability for ankle plantarflexion.
- Hip and knee ROM demonstrated limited validity but showed moderate to good inter-method reliability between in-person and online assessments.

**What are the main findings?**

- The validity of the sensor-based mobile application is confirmed only for ankle plantarflexion.
- Sensor-based applications show comparable measurements when applied in-person and online for most lower-limb joints.

**What are the implications of the main findings?**

- PhysioMaster has the potential to be used for monitoring changes in ROM online due to ease of use and consistent numbers in person and online.
- Smartphone sensor-based tools like PhysioMaster offer promising support for remote musculoskeletal assessment for longitudinal ROM tracking.

**Abstract:**

The increasing use of telerehabilitation has intensified the need for validated smartphone sensor-based tools capable of accurately capturing joint range of motion (ROM). This study examined the criterion validity of the PhysioMaster application compared with a universal goniometer during in-person assessments and evaluated the inter-method reliability between in-person and online PhysioMaster measurements. Thirty healthy young adults underwent standardized hip, knee, and ankle ROM testing using both approaches. The criterion validity was limited for most joints, with only ankle plantarflexion demonstrating the highest validity and dorsiflexion showing a moderate association; in contrast, hip and knee ROM exhibited poor agreement with goniometric values. Despite limited absolute agreement, PhysioMaster demonstrated moderate to good inter-method reliability for hip and knee ROM, indicating consistency across assessment modes. These findings suggest that while PhysioMaster may not serve as a direct substitute for in-person goniometry, it shows potential as a consistent tool for tracking ROM changes remotely, particularly for hip and knee movements. The application may support remote musculoskeletal monitoring within telerehabilitation contexts where repeated, standardized assessments are required.

## 1. Introduction

Joint range of motion (ROM) assessment is essential for musculoskeletal evaluations to inform diagnosis, determine prognosis, and monitor therapeutic progress [1]. The universal goniometer remains the clinical standard for ROM measurement [2,3]. However, reliance on manual alignment, operator skill, and in-person assessment limits its scalability [4,5]. As such, this model poses a significant barrier for individuals who face challenges in attending regular clinical visits due to movement limitations, disease severity, or transportation barriers, which often result in missed rehabilitation sessions and delayed recovery [6,7].

Advancements in digital health and mobile-sensing technologies have facilitated ROM quantification using motion-captured systems, smartphone-embedded inertial sensors, and camera-based angle estimation [4]. These tools offer cost-effective, portable, and repeatable measurement capabilities, with the potential to reduce operator-dependent variability and support continuous monitoring both within and beyond clinical environments [8,9,10]. The rapid expansion of telerehabilitation in response to healthcare access needs has further demonstrated the feasibility of remote assessment models [11]. Evidence suggests that remotely assessed ROM measurements are feasible and may achieve accuracy comparable to that of in-person methods [12,13]. Moreover, incorporating ROM assessment into telerehabilitation models may result in fewer in-person clinical visits and reduced demands on healthcare resources and services [14].

Lower-limb joints are of particular interest in telerehabilitation due to the high prevalence of knee, ankle, and hip disorders in clinical populations, as well as their critical role in mobility and functional independence [15]. While emerging research has demonstrated the good intra- and inter-rater reliability and strong validity of smartphone-based applications for spinal ROM [16,17] and upper-limb assessments [18,19], evidence supporting mobile sensor-based applications for lower-limb ROM remains limited in scope. Existing studies have primarily focused on selective ROM movements, such as hip ROM [20], straight leg raise, ankle dorsiflexion, and hip internal rotation [21], with more recent work examining knee extension only [22]. Collectively, these findings indicate a need for further research providing a comprehensive evaluation of the criterion validity of sensor-based applications across all lower-limb joints [23]. In addition, few studies have examined the agreement between in-person and remote assessment conditions, two domains crucial for the scalability of telerehabilitation [12,13,14].

Current smartphone-based applications employ diverse technologies, including utilizing users’ phone built-in sensors with guiding prompts (e.g., the PeerWell mobile application), as well as image- and video-based approaches that estimate joint angles from visual inputs (e.g., the DrGoniometer application) [23]. Other technologies include wearable inertia sensors [24] and hybrid approaches that combine sensor- and camera-derived data, all of which have been proposed for smartphone-based ROM assessment [24,25]. While many of these applications offer improved portability and accessibility, their clinical performance varies across joints, tasks, and assessment contexts [26,27].

The limitations of current models include restricted transferability to remote monitoring; for example, image- and video-based approaches may be sensitive to camera positioning, lighting conditions, and movement plane alignment [21]. In addition, current smartphone gyroscope-based tools (e.g., iPhone built-in sensors) often require extra assistance to support proper positioning and data acquisition during assessment [21]. Furthermore, applications such as PeerWell and TelePhysio were developed for use within specific healthcare systems, which may limit their generalizability and public availability [13,20]. Other technologies evaluated primarily in research settings, including RSQ motion systems [26], wearable inertial sensor platforms such as HumanTrak [24], and specialized camera-based systems [12], often require controlled environmental conditions, potentially limiting their transferability to routine clinical practice and remote assessments [12,22].

The PhysioMaster smartphone application provides a free, publicly available, sensor-based approach that leverages inertial measurement units embedded in standard smartphones to quantify ROM without the need for external hardware. Unlike more complex motion analysis systems, it is designed for rapid deployment using a single mobile device, supporting implementation in both clinical and home-based contexts [23]. Previous studies have demonstrated acceptable validity and reliability for cervical ROM assessment and knee extension for in-person assessment [17,21]. However, its performance for comprehensive lower-limb ROM measurement, particularly across in-person and remote settings, remains insufficiently characterized.

Therefore, this study investigated the criterion validity and inter-method reliability of PhysioMaster for measuring lower-limb ROM in both in-person and remote environments. We hypothesized that the PhysioMaster application would exhibit high criterion validity compared with standard goniometric measurements, as well as strong inter-method reliability for ROM measurements conducted in both in-person and remote settings. By integrating mobile-sensing technology with clinical musculoskeletal evaluation, this research aims to advance sensor-enabled rehabilitation assessments and provide evidence for an accessible, scalable, and clinically robust digital monitoring tool.

## 2. Materials and Methods

### 2.1. Study Design

**Figure 1:**
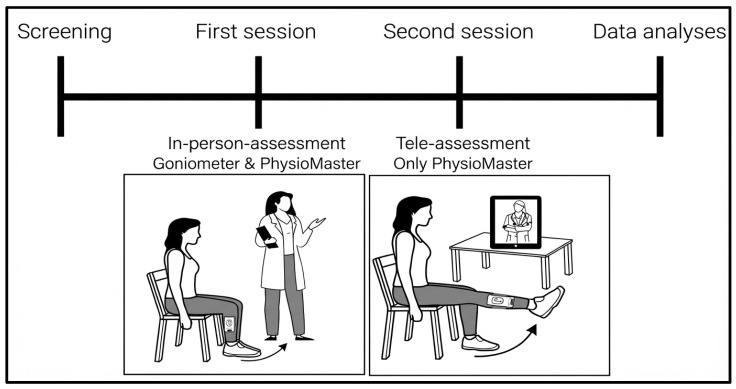
A flowchart of the study design illustrating the stages of this study.

This study employed a two-session repeated-measures design to evaluate (1) the criterion validity of the PhysioMaster smartphone application (TrinusLab, PhysioMaster, ver. 2.50, App Store, 2025, Omišalj, Croatia; https://trinuslab.com/, accessed on 1 September 2025) compared with a standard goniometer during in-person assessment (Session 1) and (2) the inter-method reliability of the application when administered remotely in a tele-assessment environment (Session 2). Only the dominant lower limb was assessed to ensure consistency across sessions and minimize inter-limb variability. The study flowchart is presented in Figure 1.

### 2.2. Participants

The required sample size was calculated using G*Power software (Version 3.1; University of California, Los Angeles, CA, USA). Assuming a moderate association (*ρ* = 0.50) and a target reliability for ROM assessment (intraclass correlation coefficient [ICC] = 0.75), an α level of 0.05, and a power of 80%, a minimum of 27 participants was necessary. To account for the 10% attrition rate, a total of 30 participants were recruited [17]. Participants were recruited based on defined eligibility criteria. The inclusion criteria specified that participants must be healthy male and female adults aged 18–30 years. Individuals with recent injuries to the spine or lower limbs, musculoskeletal disorders, neurological conditions, or cognitive impairments were excluded to ensure that all ROM measurements accurately reflected typical, non-pathological joint performance. Ethical approval was obtained from the Institutional Review Board of Majmaah University (Protocol #MUREC-May.14/COM-2025/146), and all participants provided written informed consent before participation.

### 2.3. Procedures

#### 2.3.1. Session 1: In-Person Laboratory Assessment

Standardized ROM assessments were conducted for the hip, knee, and ankle on the dominant lower limb, as defined subjectively by the participant. All measurements were performed by trained research personnel in accordance with standardized clinical protocols. For each movement, two measurements were initially obtained using a standard goniometer, followed by two measurements using the PhysioMaster application to establish baseline values and facilitate direct comparison between the two tools. The smartphone was reoriented before each trial to ensure consistent sensor alignment. This session was conducted under controlled conditions in the Physical Therapy Laboratory of the Physical Therapy Department at Majmaah University (Al Majmaah, Riyadh, Saudi Arabia).

Hip flexion and extension were assessed with participants standing beside a wall for support. For hip flexion, the goniometer was aligned with the greater trochanter, and the smartphone was secured with a strap on the anterior thigh; placement sites were marked to ensure consistency across trials. For hip extension, the device was positioned laterally on the thigh. Knee flexion and extension were assessed with participants seated on an armless chair. The goniometer was aligned with the lateral condyle, and the smartphone was positioned laterally just below the knee joint. Ankle dorsiflexion and plantarflexion were measured with participants seated and the tested leg crossed over the opposite knee; the goniometer was aligned with the medial malleolus, while the smartphone was secured medially on the foot [2].

These positions were selected because they offer stable and repeatable alignment for both goniometric and smartphone-based measurements and can be easily replicated during remote tele-assessments. For the in-person assessments, the goniometer was utilized first as the clinical reference tool, ensuring standardized and unbiased baseline measurements. All assessments were conducted twice using both instruments [17,28].

#### 2.3.2. Session 2: Remote Tele-Assessment

**Figure 2:**
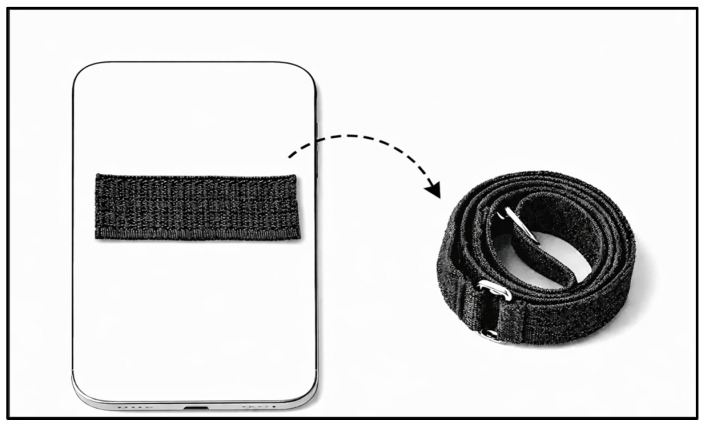
Smartphone attachment strap used for limb fixation during range of motion assessment. A Velcro patch was adhered to the posterior surface of the smartphone, allowing a detachable Velcro strap to secure the device circumferentially around the limb. The length of the strap was pre-determined based on the limb circumference during the in-person session.

Before the remote session, participants received instructions for installing the PhysioMaster application and measurement procedures (provided in the Supplementary Material). Participants were also provided with a Velcro strap that was used to secure the smartphone, with the distal end bonded to the device to permit the smartphone attachment to the participant’s limb regardless of clothing (Figure 2).

During the remote assessment, participants were instructed to repeat the same ROM measurements at home using only the PhysioMaster application. The application utilizes the inertial sensors embedded within the user’s smartphone and does not rely on camera or video tracking. However, the remote session was supervised in real time via Google Meet (Google LLC, Mountain View, CA, USA), a secure videoconferencing platform, allowing the research team to guide the procedure, verify smartphone placement, and monitor movement quality. No recordings were stored, and participant privacy was upheld throughout the assessment. The remote session was conducted within 48 h of the in-person assessment in a quiet, well-lit environment. Environmental variations were accounted for by adjusting the camera angle as needed to ensure proper visualization. Participants were asked to use a chair used at home that allowed the same arch of motion as in laboratory setting. This design enabled evaluation of the application’s performance under both controlled in-person conditions and a real-world tele-assessment environment.

**Figure 3:**
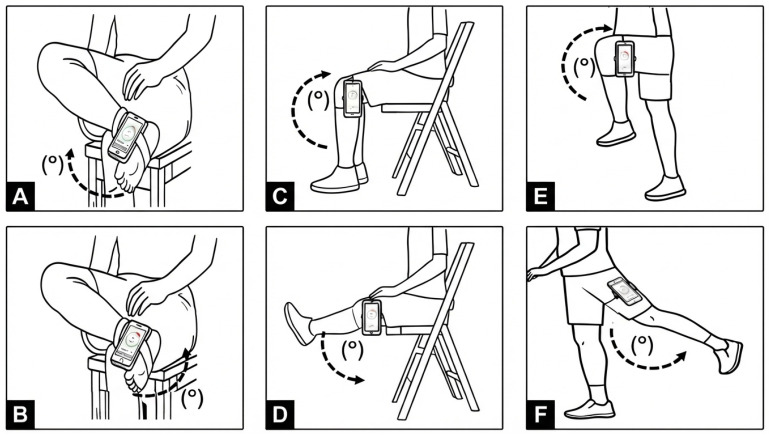
Schematic illustration of smartphone placement and measurement positions for lower-limb range of motion (ROM) assessment. Panel (**A**): ankle dorsiflexion; Panel (**B**): ankle plantarflexion; Panel (**C**): knee extension; Panel (**D**): knee flexion; Panel (**E**): hip flexion; Panel (**F**): hip extension. Dashed arcs and degree symbols (°) denote the angular displacement measured as joint ROM.

After confirming proper device positioning via real-time supervision, the examiner instructed participants to execute each movement while providing corrective feedback to minimize compensatory strategies. For each trial, participants pressed “Start,” performed the movement to their end range, and then pressed “Stop.” Two trials were conducted for each motion, and participants verbally reported the recorded values to the examiner for documentation. A schematic illustration of the positions and smartphone placement is presented in Figure 3.

### 2.4. Data and Statistical Analyses

The participants’ demographics are summarized using the mean ± standard deviation. Reliability analyses were selected based on their robustness in handling non-parametric data due to the non-normal distribution of the ROM data, as confirmed by the Shapiro–Wilk test. Interclass coefficient analysis (ICC)s are reported along with 95% confidence intervals (CIs), as they are appropriate and stable under non-normal conditions. The ICC values indicated reliability levels classified as poor (<0.50), moderate (0.50–0.75), good (0.75–0.90), or excellent (>0.90) [29]. Bland–Altman plots were generated to examine mean differences and the LOA between methods. Spearman’s rank correlation coefficient (*ρ*) was calculated to complement the ICC by assessing the monotonic relationship between measurements, with associations classified as poor (<0.30), moderate (0.30–0.60), moderately high (0.61–0.85), and high (>0.85) [30]. For correlation analyses, statistical significance was defined as two-tailed *p*-values of <0.05. Outliers exceeding ±2 standard deviations from the mean were removed at the pair level (goniometer–mobile or mobile–mobile) to preserve the integrity of paired analyses before statistical testing. Following outlier detection, 16 observations were excluded from the total dataset, comprising 30 participants with six average measurements per session for both criterion validity and inter-method reliability. These included 10 criterion validity observations (three ankle dorsiflexion, three hip extension, two knee flexion, one knee extension, and one ankle plantarflexion) and six inter-method reliability observations (three ankle dorsiflexion, one hip flexion, one hip extension, and one knee extension). All statistical analyses were conducted using SPSS Statistics (version 31; IBM Corp., Armonk, NY, USA).

## 3. Results

Thirty participants (10 men and 20 women; mean age: 20.8 ± 2.1 years; all right-leg dominant) were included in the final analysis. All participants successfully completed both the in-person and tele-assessment sessions, with no adverse events reported. Descriptive statistics for all lower-limb ROMs across both sessions are presented in Table 1.

### 3.1. Concurrent Validity (Goniometer vs. Sensor-Based Mobile Application)

The ICC values indicated that the goniometer and PhysioMaster application demonstrated significant moderate agreement only for ankle plantarflexion (ICC = 0.76; 95% CI: 0.51 to 0.89; *p* < 0.001). All other ROM measures exhibited poor to low–moderate agreement, including ankle dorsiflexion (ICC = 0.37; 95% CI: −0.36 to 0.71; *p* = 0.11), knee flexion (ICC = −0.23; 95% CI: −1.64 to 0.43; *p* = 0.70), knee extension (ICC = −0.018; 95% CI: −1.08 to 0.50; *p* = 0.520), hip flexion (ICC = −0.045; 95% CI: −1.20 to 0.50; *p* = 0.50), and hip extension (ICC = 0.097; 95% CI: −0.98 to 0.58; *p* = 0.39). Negative ICC values reflect measurement variability exceeding between-participant variability, indicating poor agreement.

Spearman’s correlation coefficients indicated a moderately strong association between measurements derived from the goniometer and PhysioMaster application for ankle plantarflexion (*ρ* = 0.64; *p* < 0.001). In contrast, the other ROM measures exhibited weak, non-significant correlations, including ankle dorsiflexion (*ρ* = 0.24; *p* = 0.22), knee flexion (*ρ* = 0.08; *p* = 0.67), knee extension (*ρ* = 0.19; *p* = 0.30), hip flexion (*ρ* = −0.08; *p* = 0.65), and hip extension (*ρ* = 0.17; *p* = 0.39).

**Figure 4:**
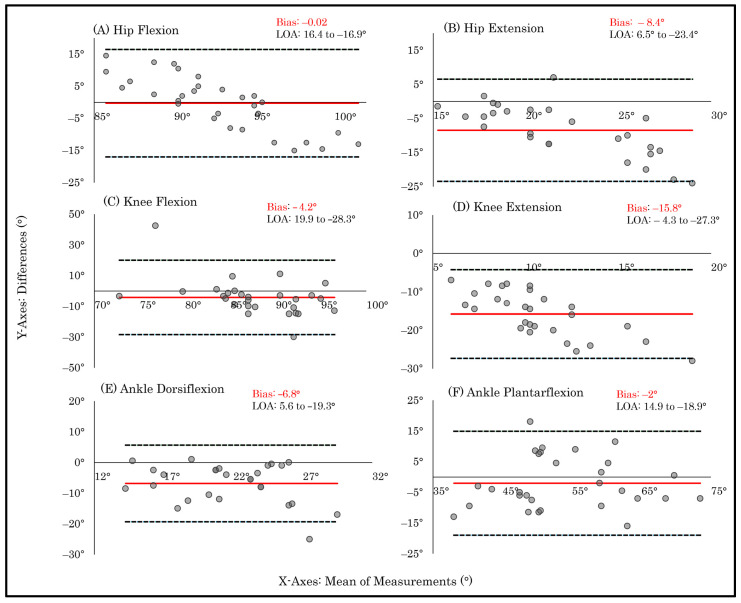
Bland–Altman plots illustrating the agreement between the two ROM measurement techniques during Session 1. The plots display the mean difference (bias) and the 95% limits of agreement (LOA) between in-person goniometric and PhysioMaster measurements of lower-limb range of motion. Individual panels show (**A**) hip flexion, (**B**) hip extension, (**C**) knee flexion, (**D**) knee extension, (**E**) ankle dorsiflexion, and (**F**) ankle plantarflexion. The central red line represents the bias, and the dashed lines represent the upper and lower LOA (±1.96 SD).

Bland–Altman plots corroborated these findings: ankle plantarflexion demonstrated comparatively lower dispersion around the mean difference. In contrast, ankle dorsiflexion, knee flexion, knee extension, hip flexion, and hip extension displayed wider limits of agreement (LOA) and greater scatter, indicating increased individual-level variability and corresponding with their lower ICC values and weak or non-significant correlations (Figure 4).

### 3.2. Inter-Method Reliability (In-Person vs. Remote Application)

The PhysioMaster application demonstrated moderate-to-good reliability for several lower-limb ROM assessments when comparing the results of in-person and remote evaluations. Significant ICC values indicated good reliability for hip flexion (ICC = 0.81; 95% CI: 0.61 to 0.91; *p* < 0.001) and knee flexion (ICC = 0.76; 95% CI: 0.50 to 0.89; *p* < 0.001). Moderate reliability was observed for hip extension (ICC = 0.67; 95% CI: 0.31 to 0.85; *p* = 0.002) and ankle plantarflexion (ICC = 0.61; 95% CI: 0.17 to 0.82; *p* = 0.008), although the wide confidence intervals indicate notable variability. In contrast, knee extension (ICC = 0.41; 95% CI: −0.25 to 0.72; *p* = 0.08) and ankle dorsiflexion (ICC = 0.14; 95% CI: −0.83 to 0.59; *p* = 0.34) demonstrated poor and non-significant reliability across assessments.

**Figure 5:**
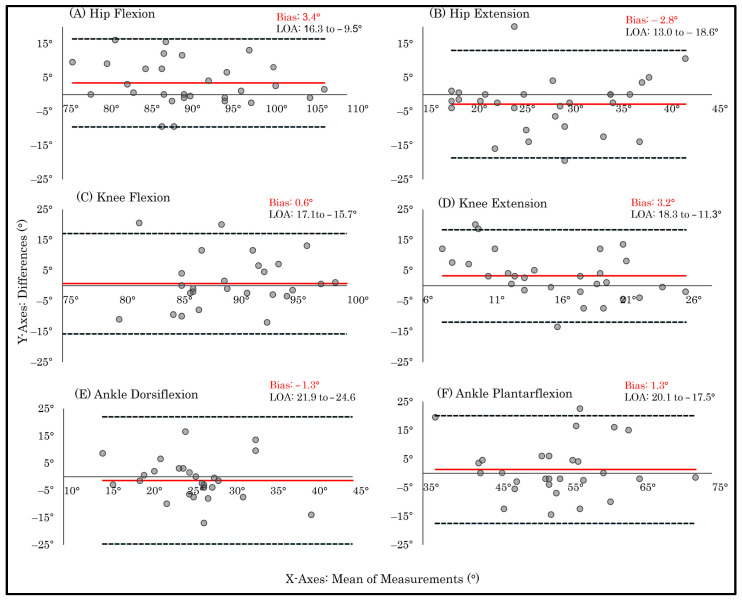
Bland–Altman plots illustrating the agreement of PhysioMaster measurements obtained under two conditions: (1) in-person assessment and (2) remote assessment during Session 2. The plots display the mean difference (bias) and the 95% limits of agreement (LOA) for lower-limb range of motion. Individual panels present (**A**) hip flexion, (**B**) hip extension, (**C**) knee flexion, (**D**) knee extension, (**E**) ankle dorsiflexion, and (**F**) ankle plantarflexion. The central red line represents the bias, and the dashed lines indicate the upper and lower LOA (±1.96 SD).

Spearman’s correlation coefficients showed a strong and statistically significant association for hip flexion (*ρ* = 0.67; *p* < 0.001). Moderate and statistically significant associations were observed for hip extension (*ρ* = 0.58; *p* < 0.001), knee flexion (*ρ* = 0.46; *p* = 0.01), and ankle plantarflexion (*ρ* = 0.44; *p* < 0.02). In contrast, knee extension (*ρ* = 0.36; *p* = 0.54) and ankle dorsiflexion (*ρ* = 0.17; *p* = 0.36) demonstrated weak, non-significant correlations, indicating poor agreement between in-person and remote assessments for these movements. Bland–Altman analysis revealed wide LOA across all lower-limb ROM measures. Notably, knee extension and ankle dorsiflexion exhibited disproportionately greater dispersion of differences relative to their respective ROMs, alongside wider LOA, reflecting reduced measurement stability (Figure 5).

## 4. Discussion

This study evaluated the criterion validity and inter-method reliability of the PhysioMaster smartphone application for measuring lower-limb ROM compared with a standard goniometer during both in-person and remote assessments in healthy young adults. The results demonstrate joint-dependent variability in measurement performance. Although moderate agreement was observed for selected movements, several joints showed poor concordance across assessment modes. These findings indicate that the accuracy of smartphone-based ROM assessment is not uniform across joints and may be influenced by joint-specific anatomical complexity and assessment conditions.

In terms of criterion validity, ankle plantarflexion exhibited the strongest concordance with goniometric measurements, whereas ankle dorsiflexion, hip and knee ROMs displayed weaker or inconsistent concordance. This pattern reflects the heterogeneous performance of the PhysioMaster application across ROM measures, resulting in joint-specific variability in criterion validity. These findings align with previous studies indicating that smartphone-based measurement tools demonstrate stronger agreement for selected ROM measures than for others, suggesting that their clinical utility may vary depending on the joint and movement assessed [16,22].

Inter-method reliability between in-person and remote assessments also demonstrated a movement-dependent pattern. Concordance between assessment methods was strongest for hip flexion, hip extension, knee flexion, and ankle plantarflexion, whereas knee extension and ankle dorsiflexion showed minimal agreement. The stronger inter-method reliability observed for larger ROMs likely reflects the PhysioMaster application’s ability to preserve measurements with more easily identifiable end-range positions. In contrast, smaller ROMs and less distinct visual endpoints, such as knee extension and ankle dorsiflexion, were more susceptible to variability. This variability was likely exacerbated by user-dependent factors and subtle differences in smartphone positioning. These findings align with prior evidence suggesting that remote ROM assessment is feasible when participant positioning and smartphone alignment are standardized; however, even minor deviations can significantly impact measurement precision [23,24].

The contrasting patterns observed between criterion validity and inter-method reliability highlight important measurement considerations. Several ROM measures demonstrated moderate inter-method reliability but low criterion validity relative to the goniometric reference, indicating internal consistency without close alignment to gold-standard values. Conversely, ROM measures that showed greater criterion alignment under controlled conditions remained sensitive to environmental variation during remote assessments. Across analyses, ankle plantarflexion consistently demonstrated the highest performance, likely due to joint-specific anatomical characteristics, reduced soft-tissue artifact, and its larger ROM relative to dorsiflexion. Larger angular displacements may be easily captured by inertial sensors, thereby enhancing measurement sensitivity and agreement.

Bland–Altman analyses revealed relatively small mean biases across most ROM measures, indicating that PhysioMaster does not systematically overestimate or underestimate joint ROM. However, wide LOA were observed across all joints, reflecting substantial individual-level variability across both goniometer application and in-person–remote application comparisons, indicating limited interchangeability between the PhysioMaster application and the goniometer. Although ankle plantarflexion exhibited comparatively lower variability than other movements, its LOA remained wide, reinforcing this lack of interchangeability. When interpreted relative to reported minimal detectable change thresholds (approximately 5°–10°) [31], the observed LOA may exceed clinically acceptable measurement error margins relevant for rehabilitation decision-making [24]. Consequently, while remote assessments may approximate in-person measurements at the group level, individual measurements may differ sufficiently to influence clinical decisions. These findings suggest that remote ROM assessment may be more suitable for monitoring trends or repeated measurements over time rather than for single-point, threshold-based decisions, such as determining discharge readiness, or treatment modification based on predefined ROM measurements cutoffs [27].

From a telerehabilitation perspective, these findings emphasize the role of PhysioMaster as a complementary tool rather than a replacement for traditional goniometric assessment. While its accuracy varies across joints, its cross-platform accessibility may support remote follow-up and continuity of care for individuals with limited access to in-person therapy sessions. However, the joint-specific limitations observed in this study highlight the need for continued methodological refinement, including improved standardization of sensor placement and environmental control. This interpretation is consistent with previous findings indicating that acceptable accuracy in remote ROM measurement depends heavily on operational control, while device- and environment-related variability remain key limiting factors [12,13,14].

Such results may, in part, reflect the technical mechanism of the PhysioMaster application. The application relies on smartphone inertial sensors (e.g., gyroscope and accelerometer) rather than camera-based tracking, and measurement accuracy may therefore vary across devices due to differences in sensor specifications and algorithmic processing. As these technical details were not disclosed by the developers, a clearer understanding of device- and algorithm-related factors may help explain the reduced agreement observed for certain lower-limb joints. This variability may be further exacerbated in remote assessment contexts, where participants use personal devices. Although measurement accuracy does not yet match that of gold-standard tools across all joints, these findings align with broader evidence supporting the role of remote assessment tools in telerehabilitation, while also underscoring the need for further refinement before clinical interchangeability can be achieved [22,26].

This study has certain limitations that warrant acknowledgment. First, the sample consisted of healthy young adults, who are likely to demonstrate consistent ROM and high protocol compliance, potentially optimizing application performance and limiting generalizability to older or clinical populations [32]. Second, measurements were conducted solely on the dominant limb, which may demonstrate more optimized movement patterns and reduce variability, thereby underestimating inter-limb differences. Third, although smartphone placement, monitoring quality, and allowance of ROM arcs within participants’ environments were controlled, several technical and environmental factors were not standardized across home settings. These included the type of smartphone used (e.g., variations in device size, and weight), camera height and angle, chair dimensions, and clothing worn during assessments. These factors may have introduced additional variability in remote ROM measurement.

Additionally, the absence of inter- and intra-rater reliability analyses limits the interpretability of the findings. These limitations are consistent with challenges reported in prior telerehabilitation research [24,27]. Concurrently, emerging literature continues to expand the use of smartphone-based applications for functional assessment [33,34] and their translation into clinical practice [35]. Future studies should extend criterion validity analyses by incorporating inter- and intra-rater reliability and evaluating automated guidance systems within telerehabilitation contexts. In parallel, further methodological advances in smartphone-based applications may benefit from automated camera alignment guidance, real-time feedback on limb positioning, and three-dimensional computer vision-based landmark detection. In addition, systematic evaluation of emerging sensor technologies and optimized tracking algorithms may enhance measurement precision, clinical utility, and applicability across both healthy and clinical populations in telerehabilitation settings.

## 5. Conclusions

The PhysioMaster application exhibited movement-dependent performance in lower-limb ROM assessment, demonstrating the strongest criterion validity for ankle plantarflexion. Inter-method reliability was moderate for selected movements, including hip flexion, hip extension, knee flexion, and ankle plantarflexion. These findings suggest that PhysioMaster has the potential to serve as an adjunct tool for remotely monitoring ROM, particularly for movements characterized by larger arcs or distinct anatomical landmarks. However, the wide LOA observed across all joints indicates substantial individual-level variability, limiting the interchangeability of PhysioMaster measurements with traditional goniometric assessments for precise individual evaluations.

## Figures and Tables

**Table 1:** Descriptive lower-limb range of motion measurements obtained using goniometer and PhysioMaster during in-person assessment (Session 1) and remote PhysioMaster assessment (Session 2). Data are presented as median (interquartile range, IQR). IQR was calculated as the difference between the 75th and 25th percentiles. All values represent joint angles in degrees (°).

Joint Movement	Session 1 (In Person)		Session 2 (Remote)
	Goniometer	PhysioMaster	PhysioMaster
Hip flexion (°)	92.5 (4.9)	92.3 (9.8)	90.5 (14)
Hip extension (°)	17.5 (4)	25.5 (12)	31 (14)
Knee extension (°)	2.5 (4)	18.3 (6.3)	14.5 (9)
Knee flexion (°)	85 (7.3)	91.9 (10.5)	90 (9.5)
Ankle dorsiflexion (°)	19.5 (5.5)	25.5 (4.5)	28 (9.5)
Ankle plantarflexion (°)	54.5 (15)	53 (13.5)	53.5 (9)

## Data Availability

The data created in this manuscript will be shared upon appropriate request from the corresponding author.

## Supplementary Materials

The following supporting information can be downloaded at: https://www.mdpi.com/article/10.3390/s26051661/s1.
