# Supplementary figures and images for: Criterion Validity and Inter-Method Reliability of a Smartphone Sensor-Based Application for Lower-Limb Range of Motion: In-Person vs. Tele-Assessment
Source: Sensors (Basel). 2026 Mar 6;26(5):1661. doi: 10.3390/s26051661 (PMC12987158; doi:10.3390/s26051661)

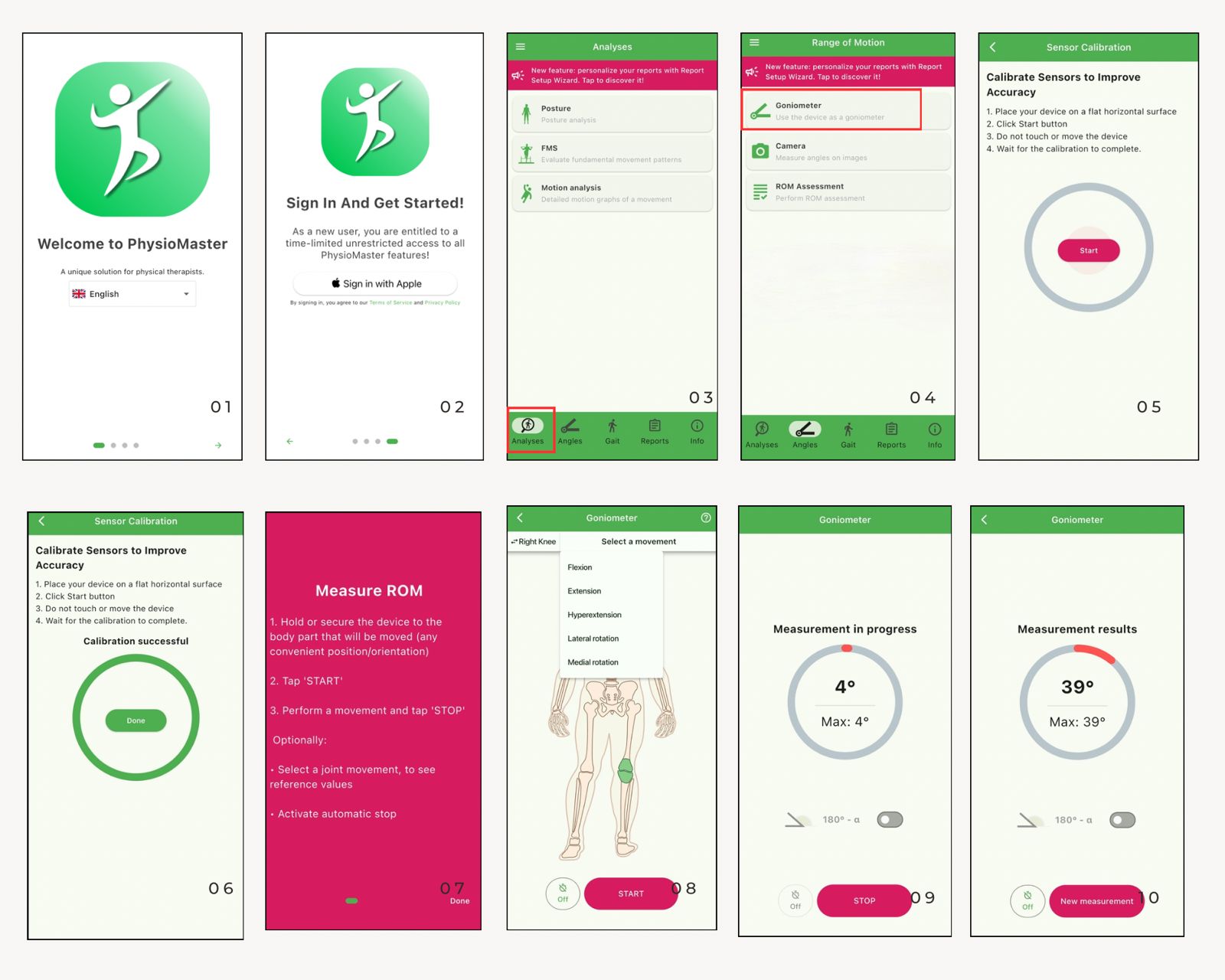

Supplement: Supplementary file 1 [file sensors-26-01661-s001.zip › sensors-4125717-supplementary.jpeg]
